# Secondary Antibiotic Resistance, Correlation between Genotypic and Phenotypic Methods and Treatment in *Helicobacter pylori* Infected Patients: A Retrospective Study

**DOI:** 10.3390/antibiotics9090549

**Published:** 2020-08-28

**Authors:** Maria Teresa Mascellino, Alessandra Oliva, Maria Claudia Miele, Massimiliano De Angelis, Giovanni Bruno, Carola Severi

**Affiliations:** 1 Department of Public Health and Infectious Diseases, Sapienza University, 00185 Rome, Italy; alessandra.oliva@uniroma1.it (A.O.); mariaclaudia.miele@uniroma1.it (M.C.M.); massimiliano.deangelis@uniroma1.it (M.D.A.); 2 Department of Translational and Precision Medicine, Sapienza University, 00185 Rome, Italy; giovanni.bruno@uniroma1.it (G.B.); carola.severi@uniroma1.it (C.S.)

**Keywords:** *Helicobacter pylori* infection, molecular methods, E-test, multiresistance, patients therapy

## Abstract

The aim of this study was to evaluate the secondary resistance in *Helicobacter pylori (Hp)* infected patients who had failed a first-line therapy, and to compare the genotypic tests performed directly on gastric samples with phenotypic tests performed on culture media. The eradication rate of patients treated with bismuth quadruple therapy (BQT) is also evaluated. A total of 80 positive specimens were retrospectively examined. Antibiotic susceptibility testing of *Hp* strains was performed by E-test, whereas a molecular commercially available method was used for detecting the mutations involved in clarithromycin and levofloxacin resistance. High resistance levels to metronidazole and clarithromycin (61.6% and 35%, respectively) and worrying resistance levels to levofloxacin (15%) were found phenotypically. Multiple resistance to two or three antibiotics was observed as well. The polymorphism A2143G on clarithromycin 23S rRNA gene was found in 34/80 (42.5%) isolates including 10 mixed infections (29%), whereas 28/80 (35%) strains were resistant phenotypically. Levofloxacin resistance corresponded to 30% by PCR and 15% by E-test (statistically significant, *p* < 0.05). The knowledge of clarithromycin and levofloxacin resistance is crucial to establish an appropriate therapy in different geographical areas. The genetic methods were superior to phenotypic techniques in the absence of live bacteria or for identifying mixed infections that may lead to a resistance underestimation. The BQT eradication rate was effective (90%).

## 1. Introduction

The *Helicobacter pylori (Hp)* infection is one of the most common chronic bacterial disease in humans. It plays an important role in different pathologies, such as chronic active gastritis, peptic ulcer disease, gastric carcinoma, mucosa-associated lymphoid tissue (MALT) lymphoma, and endothelial dysfunctions leading to vascular diseases [1]. The antibiotics once regarded as the first choice for *Hp* infection (such as metronidazole and clarithromycin), included in all therapeutic regimens, are now declining in efficacy because of their extensive use in many areas for unrelated infections. Metronidazole mostly showed a very high resistance worldwide achieving a level up to 78.2% in China [2]. In developing areas, such as in Bangladesh where the prevalence of *Hp* infection among the population ranges from 84% in children to 92% in adults, resistance rates to metronidazole reached high levels (77.5%), as well as those to tetracycline, and amoxicillin (15% and 6.6%, respectively). In this country, the knowledge of antimicrobial resistance in *Hp* is still lacking and this issue represents a big problem [3].

The resistance patterns of *Hp* isolated in our country exhibit complicated antibiotic resistance features, especially in southern Italy [4,5]. The local susceptibility and the clarithromycin-resistance levels are crucial to establish a correct therapy. Clarithromycin resistance rates have currently reached high levels, such as 30% in Italy and Japan, 40% in Turkey, and 50% in China, although rates in Sweden, Taiwan, and Germany are lower (<15%) [6]. In a study conducted in central Germany, Selgrad et al. [7] found that primary, secondary, and tertiary resistances against clarithromycin were 7.5%, 63.2%, and 75.4%, respectively—highlighting the relationship between antibiotic resistance and the previous number of eradication therapies over a period of seven years thereby observing a great increase of *Hp* antibiotic resistance in this country. In the same study, the primary, secondary, and tertiary resistances to metronidazole were 32.7%, 63.2%, and 80.1%, respectively confirming the high level of resistance to metronidazole.

Fluoroquinolone resistance has been increasing worldwide in recent years achieving 20% in Italy, 13.3% in Germany, and 19.2% in China [2,8]. These data are especially important in those regions planning a levofloxacin-based therapy because resistance to fluoroquinolones generally shows a major impact on the success of treatment [9]. Levofloxacin is generally used as a second-line drug and is recommended only after susceptibility testing, due to its high secondary resistance [10]. The primary, secondary, and tertiary resistance to levofloxacin was reported to be 11.7%, 17.6%, and 36.4%, respectively [7].

Antibiotic resistance is usually identified by phenotypic methods through the measurement of MIC (Minimum Inhibitory Concentration) values [11]. However, in those antibiotics, such as clarithromycin and levofloxacin, where the resistance is mostly acquired through point mutations (on the bacterial 23S rRNA and on the *gyrA* genes, respectively), resistance can be detected by molecular methods for easy and simultaneous detection of frequent point mutations responsible for resistance [10,12].

Aim of our research was to study retrospectively a group of patients (80) with upper gastritis who failed a previous first-line treatment, UBT (Urea Breath Test) positive, undergone an endoscopy in an Academic Hospital in Rome (Italy). We studied the secondary resistance of *Hp* to the most common antibiotics (metronidazole—MZ, clarithromycin—CLA, levofloxacin—LEV, amoxicillin—AMX, and tetracycline—TE) by focusing our research on the correlation between phenotypic susceptibility (MIC results) of *Hp* grown by culture and the genotypic susceptibility to CLA and LEV directly from gastric biopsies through molecular methods. The eradication rate of patients treated with bismuth quadruple therapy (BQT) was also evaluated.

## 2. Results

### 2.1. Demographic Data of Patients

Eighty subjects (54 women and 26 men, mean age 59 years old, range 46–82 years) infected by *Hp* who had failed a first-line therapy, were selected in our hospital and retrospectively analyzed. Familiarity with *Hp* infection was found in 66.6% of the patients, whereas smoking habit was highlighted in 20% and the use of NSAID (non-steroidal anti-inflammatory drugs) and the alcohol consumption in 25% and 30%, respectively.

As far as the endoscopic findings of the 80 positive individuals are concerned, 27 (33.4%) showed a peptic ulcer disease (seven with active ulcer and 20 with healed ulcer scar), 49 (61.6%) showed a non-ulcer disease and four (5%) had intestinal erosions. As for the histological findings, chronic gastritis was found in 60 patients (75%), severe gastritis in 16 (20%) and intestinal metaplasia in four (5%).

### 2.2. Overall Phenotypic Hp Antibiotic Resistance

From the eighty patients taken into account, we isolated by the biopsies culture of the stomach antrum of each patient eighty strains of *Hp* that were subjected to the phenotypic susceptibility test. For each sample, the MIC was determined by E-test and resistance was calculated following the breakpoints recommended by EUCAST 2020 (European Committee on Antimicrobial Susceptibility Testing) [13]. The resistance percentage measured phenotypically through MIC values to MZ, CLA, LEV, TE and AMX resulted as follows: 61.6% (50/80), 35% (28/80), 15% (12/80), 2.5% (2/80), and 1.25% (1/80), respectively. The MICs resulted as being very high mainly to MZ showing three strains with MIC = 256 µg/mL and to CLA showing four strains with MIC ranging from 64 µg/mL to 128 µg/mL. As for LEV only one strain showed a MIC equal to 32 µg/mL. In Table 1 the values of MIC_50_ and MIC_90_, and the MIC ranges of the tested antibiotics are reported.

**Figure 1:**
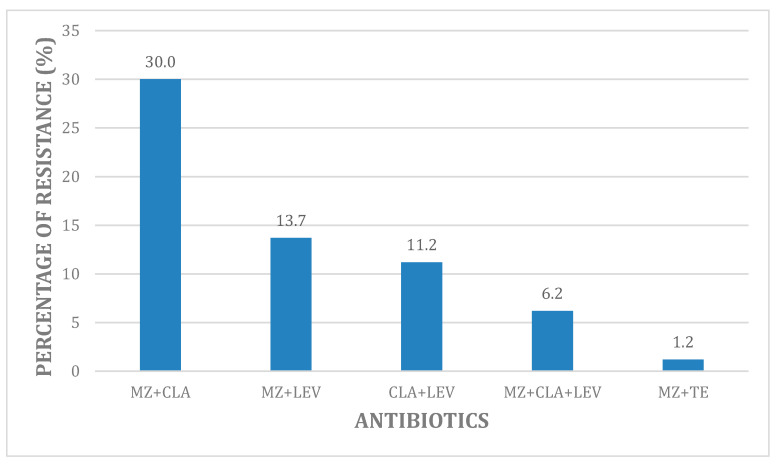
Characteristics of phenotypic antibiotic resistance for *Hp* strains. Notes: Proportion of phenotypic resistant *Hp* isolates (%) to the respective antibiotics in an Academic Hospital in Rome (Italy). Abbreviations: MZ: metronidazole; CLA, clarithromycin; LEV, levofloxacin; TE, tetracycline.

Multidrug-resistance to antibiotics is shown in Figure 1. Twenty-three samples (38.3%) carried *Hp* susceptible to all three antibiotics (CLA-MZ-LEV) (data not shown). Resistance belonging to the association CLA + MZ was the highest one being detected in 30% of samples (24/80), resistance to the combinations MEZ + LEV and CLA + LEV ranged between 13.75% (11/80) and 11.25% (9/80 strains), respectively. Only 6.25% samples (5/80) harbored the triple resistance MZ + CLA + LEV. As far as the dual combination MZ + TE is concerned, resistance was found just in one strain (1.25%). In general, we noticed that the associations consisting of MZ, CLA and LEV showed higher combined resistance rates than the antibiotic combinations recommended in BQT that show lower concomitant resistance rates as well as those reported in China [2].

### 2.3. Genetic Resistance to Clarithromycin and Levofloxacin: Correlation with In Vitro Phenotypic Method

All gastric samples underwent a genetic resistance determination to CLA and LEV. In Table 2, the molecular methods (genotypic test by PCR) and the E-test (phenotypic test) are reported. We found the polymorphism A2143G (point mutation) on CLA 23S rRNA gene conferring resistance to CLA in 34 samples out of 80 (42.5%) of which 24 only yielded an A2143G mutation, whereas 10 yielded both DNA of wild type (WT) *Hp* and mutant strains indicating a mixed infection (29.4%). Comparing these results with E-test method, we detected fewer strains showing resistance through the phenotypic method (28/80, 35%) all having MIC >0.5 µg/mL: Twenty strains were included within the group of PCR tests carrying only the mutation A2143G with MIC ranging from 4 µg/mL to 128 µg/mL, while the remaining right strains belonged to the group of mixed infections (WT + A2143G) showing lower MIC values ranging between >0.5 µg/mL and ≤4 µg/mL). As far as LEV resistance is concerned (Table 2), the *gyrA* mutations found in codon N87K (more frequent 18/24) and in codon D91G (6/24) were detected in 24 samples out of 80 (30%) by PCR method, whereas 12/80 (15%) resulted as being resistant through the phenotypic method (MIC > 1 µg/mL). Out of the 12 resistant strains by E-test, right belonged to the group of *gyrA* mutations in codon N87K (MIC = 4–32 µg/mL), whereas four belonged to the group of *gyrA* mutations in codon D91G (MIC between >1 µg/mL and ≤4 µg/mL).

Interestingly we found that higher MIC values of CLA and LEV (128 µg/mL and 32 µg/mL, respectively) were associated to strains carrying the point mutation A2143G alone for CLA and the *gyrA* mutation correlated to the codon N87K for LEV. The different detection of resistance between PCR and E-test to CLA was not statistically significant (42.5% vs. 35%, *p*-value = 0.417 then >0.05). On the contrary, this difference was statistically significant for LEV where the respective values were 30% vs. 15%, *p*-value = 0.036 then <0.05.

In addition to the above 80 strains belonging to the 80 patients under consideration, three extra specimens belonging to further three patients in whom bacterial culture showed no live bacteria, but only coccoid forms which are inactive bacteria unable to replicate, and therefore, unable to be subjected to the phenotypic antibiotic sensitivity test, were considered [14] (see Material and Methods section). However, by the genotypic methods performed on the three single gastric tissues in order to evaluate the *Hp* susceptibility to CLA and LEV, we found resistance to both antibiotics for all three samples (data not shown).

### 2.4. Eradication Rate

The eradication rate of the patients undergone for 14 days a quadruple therapy based on PPI (proton pump inhibitor: Omeprazole 20 mg), bismuth (220 mg bid), MZ (400 mg tid) and TE (250 mg tid) consistently with the high rate of CLA resistance found in our population, was 90% (72/80). A 2-week MZ use may overcome the negative influence of MZ-resistance [9,15].

## 3. Discussion

This study indicates that high degrees of resistance to antibiotics is still a challenge to the *Hp* eradication in our country. Only CLA and LEV were subjected to molecular investigations because they were considered the cornerstones in the *Hp* therapy being CLA until recently considered the most powerful antibiotic against *Hp* (even though in several countries a high resistance is detected) and being LEV a valid alternative treatment as a second-line therapy after the susceptibility tests [10,15,16]. Moreover, *Hp* showed a lower resistance rate to LEV in comparison to CLA probably due to a later addition of LEV to treatment protocols of *Hp* infection. No molecular diagnostic tests were generally performed for detecting the TE-resistance on gastric tissue samples because this may be of a little significance for initial antibiotic selection at the beginning of therapy, owing to the well documented low resistance rate to TE [4,5,6].

The prevalence of CLA resistance may induce low eradication rate, especially for sequential and concomitant therapies [9]. In contrast, the quadruple based therapy with a combination of two antibiotics (i.e., TE + MZ or AMX + MZ) is recommended because the risk of having TE or AMX resistant strains is very low and MZ-resistance is shown to have no impact on therapeutic outcomes [17]. In fact, the Bismuth Quadruple Therapy has proven high efficacy in spite of MZ resistance in Europe also bypassing the quinolone resistance. Triple therapy with CLA turns out to be obsolete in those regions where the resistance to CLA is >15%, such as Italy [4,5]. High dual resistance to CLA and MZ >15% will impair the efficacy of all non-BQT [18]. In our patients, being this dual resistance equal to 30% and following the results of the susceptibility tests, the BQT seemed to be the most appropriate treatment. The local susceptibility and the CLA-resistance levels appeared to be crucial to establish a correct therapy.

The resistance to CLA is reported to be increasing all over the world, and in some countries, it depends on the local seropositivity rate. For instance, in the People’s Republic of China, an increase of CLA resistance from 15% in 2000 to 53% in 2014 was accompanied by a rise in seropositivity rates from 65% to 83% [19]. In contrast, in a multicenter retrospective study of the child population in the People’s Republic of China, the pattern of *Hp* antibiotic resistance showed no significant changes in the resistance rates to CLA, AMX, furazolidone, and MZ over seven years [20].

In Japan, an increase in CLA resistance ranging from 1.8% in 1996 to 27.1% in 2008 has been described [21]. In Korea, CLA resistance increased from 11% in 2015 to 60% in 2019, as well as resistance to other antibiotics (MZ, LEV, TE, AMX) [22]. The only antimicrobial agents the authors found to be effective were rifabutin and furazolidone. In Morocco (Rabat), the prevalence of primary resistance to CLA was 29% with 2% of strains showing double resistance to MZ and CLA at the same time [23]. In the USA, there is an increase of CLA resistance as well. [24]. In a study on a pediatric population in the USA, the resistance rate was as high as 50% [25]. As far as LEV is concerned, there is a more limited number of studies evaluating susceptibility to LEV. In Italy, LEV resistance has been reported to be 22–24%, as well as in Portugal [26]. LEV resistance rate of 11% was detected in Morocco by Bouihat et al. [23] in a multicenter study of patients who had never been treated previously. Interestingly a recent article [27] dealing with a study in Korea reports a new mutation of *gyrA* (a novel Gly-85 mutation of *gyrA*) different from those we have examined in our research, conferring resistance to fluoroquinolones. In this case, the authors found that the resistance rates increased from 19.0% to 43.8% both for levofloxacin and moxifloxacin.

MZ shows a high rate of resistance (50–80%) in almost all the studied countries achieving a rate of 80%, especially in developing areas [2,3]. Nevertheless, in spite of its high resistance in vitro, it is included in the BQT. It can be noted that the E-test is a reliable method for measuring antibiotic resistance to all antimicrobials in vitro but, as far as MZ is concerned, it overestimates the resistance to MZ This discrepancy between in vitro MZ resistance and treatment outcome may partially be explained by changes in oxygen pressure in the gastric environment, as MZ-resistant *Hp* isolates become MZ susceptible under low oxygen conditions in vivo [28]. In Mexico the resistance rate was about 77% [29], in China 80% [2] and in Iran 66% [30]. In this last study conducted among an Iranian population the resistances to CLA, AMX, TE, ciprofloxacin (CIP), LEV and furazolidone corresponded to 22.4%, 16.0%, 12.2%, 21.0%, 5.3% and 21.6%, respectively (note the high resistance rate found towards TE and AMX compared to Western Europe).

Due to the fact that *Hp* shows multi-resistance to the common antibiotics even to two or three antibiotics at the same time, the researchers are obstinately looking for new compounds (no antibiotics) able to efficiently combat this infection. Several articles can be found in the literature. Interesting is the study performed in Pakistan, which focuses on the activity of guanidine derivatives with low molecular weight against multidrug-resistant Gram-negative or Gram-positive bacteria. A new compound, a guanidine derivative bearing adamantane-1-carbonyl 2-bromo-4,6-difluouro-phenyl substituents (H-BDF), seemed to be promising against the strains tested [31]. Other substances were studied against *Hp*, such as three known and five unknown N-substitute-2-oxo- H-1-benzopyran-3- carboxamides (coumarin-3-carboxamides). The compounds with a 4-acyl-phenyl group showed the best activity against *Hp* metronidazole-resistant strains [32]. Other solutions should be pursued in order to increase the cure rate of *Hp* infection. Non-traditional therapies have been indicated as a means to target this important gastric pathogen. For instance, the potential role of N-acetilcysteine which is capable of destroying bacterial biofilm, is an emerging treatment for recalcitrant infections. [33] or the use of vonoprazan (potassium-competitive acid blocker P-CAB), which could improve eradication rates by raising the intragastric pH, and thus, increasing bacterial antibiotic susceptibility [34].

As far as the phenotype-genotype correlation is concerned in our study, no statistically significant difference (*p* > 0.05) was found for CLA (even though more resistant strains were detected through PCR), whereas for LEV this difference was statistically significant (*p*-value < 0.05). Nevertheless the genotypic-resistance proved to be very useful in the case of absence of live bacteria (ie.when coccoid forms were detected) as well as in identifying the mixed infections which represent a real problem that could lead to a resistance underestimation. In fact, in our three extra specimens taken into account where just inactive coccoid forms were detected, only the genetics methods were able to show the resistance to LEV and CLA. This concept is clearly underlined in the literature. In several studies conducted in Germany [8,10], the predominance of genotypic methods over the phenotypic methods is hoped for evaluating the primary or secondary resistance to CLA or LEV in order to address a specific therapy in that geographical area. The comparison between the phenotypic and genotypic methods is reported by different authors in various countries. Binyamin D. et al. [35] in a research conducted in Israel, found that for levofloxacin the genotypic methods were superior to the phenotypic ones (21.2% vs. 0%, respectively, difference statistically significant), whereas for CLA this difference was not statistically significant (66.7% vs. 62.9%). These data were comparable to our results. In another research conducted in Taiwan [36] the authors found that the agreement between genotypic and phenotypic resistance to clarithromycin was best when the MIC breakpoint was >2 μg/mL, whereas the agreement between genotypic and phenotypic resistance to levofloxacin was best when the MIC breakpoint was >1 μg/mL. In t situations, a better treatment outcome was highlighted in the population. Furthermore, the reliability of the molecular test used in our research (GenoType HelicoDR) was underlined by Lee J.W. et al., in a Korean research [37], where for isolates with A2143G mutation (CLA), 83.3% of concordance rate was estimated between our test and DNA sequencing method, whereas 85.7% was estimated for MIC test. For the isolates showing N87K mutation (LEV), this concordance was 71.1% and 88.4%, respectively.

The real-time PCR detects the resistant population at a lower concentration than the phenotypic tests, which primarily show susceptible bacteria. Following our results, the E-test is unable to detect all resistant strains because when there are many susceptible bacteria, compared with the resistant ones, these susceptible bacteria are first identified—leading to a misclassification. In fact, for carrying out the E-test, the colonies from the culture media are randomly collected. This means that either susceptible or resistant colonies can be picked up and examined, but for what has been reported above, there is a greater chance of processing susceptible colonies. On the contrary, through a direct examination of gastric samples, a diversity of strains (susceptible, resistant or both) can be detected at the same time. As a matter of fact, in this situation, resistant bacteria are more difficult to be detected by E-test than by real-time PCR where mixed bacterial populations with both resistant and susceptible strains are easily identified [38]. Mixed *Hp* infections were demonstrated in patients yielding both wild type and resistant strains in a single gastric region at the same time.

The use of genotypic tests directly on clinical specimens shows another big advantage. Indeed, they could predict the antibiotic resistance addressing changes in previous treatments or evaluating the primary resistance to antibiotics (i.e., CLA) because the knowledge of primary resistance in patients’ treatment-naïve is critical in order to avoid administration of ineffective antimicrobials. In this way, it is possible to quickly identify patients not suitable for CLA or LEV-based treatments, thereby helping clinicians to choose an appropriate therapy [8].

## 4. Materials and Methods

### 4.1. Patients Selection

Eighty patients (54 women, 26 men, mean age 59 years old, age range 46–82) with *Hp* infection showing upper gastric symptoms that required assessment with diagnostic endoscopy during 2017 in an Academic Hospital in Rome (Italy), were selected based on the *Hp* culture positivity (no presence of coccoid forms) and analyzed. A total of 80 antrum biopsies taken from the 80 subjects were positive to RUT (rapid urease test) performed before sample processing [39]. Considering the high specificity and sensitivity of this test, we can affirm that all 80 individuals were infected by *Hp.* Three further subjects in addition to the previous 80, with *Hp* infection presenting only coccoid forms (no live bacteria) in culture media, were also considered and were subjected to the molecular methods on their gastric biopsies.

No approval was required by the Ethics Committee because gastroscopy was performed exclusively for diagnostic purposes. All data were analyzed anonymously. The patients were asked to sign an informed consent to undergo an esophago-gastro-duodenoscopy with multiple biopsies.

### 4.2. Culture and E-Test Susceptibility

Biopsies taken from the antrum of the patients’ stomach were collected and submitted to culture and antimicrobials testing phenotypically [40,41]. Essential conditions for *Hp* culture were the following: Microaerophilic atmosphere, the temperature of 37 °C (range 33–40 °C), presence of 0.5% glycine. The culture medium used was Pylori Selective Agar (bio-Merieux, Marcy L’Etoile, France) with 5% sheep blood and antibiotics (amphotericin, vancomycin and trimethoprim). The microorganisms were identified through the following tests: Colony morphology, characteristic spiral-shaped Gram-negative bacteria and positive findings on oxidase, urease and catalase tests [41]. *Hp* may exist in two different morphologic forms: Normal spiral bacillary which include the infective bacterium and coccoid forms that are thought to be the dormant phase which is unable to replicate and then unable to be subjected to phenotypic antimicrobial tests, but somehow able to cause gastritis [14]. The coccoid forms were detected other than by microscope observation and by the presence of very small colonies also by Giemsa and *Hp* immunohistochemical stain. The coccoid forms resulted in positive for both stains.

For our research, we selected the 80 patients with a positive culture for infective *Hp* and the three patients with the sole presence of coccoid forms whose gastric biopsies were only subjected to molecular methods, it is impossible to perform the E-test. A sub-culture of the isolated colonies of spiral bacillary form was performed in order to obtain secondary isolation used for antibiotic sensitivity tests. The bacterial inoculum for the E-test was performed picking up the colonies grown on culture media subsequently suspended in 0.9% NaCl solution to a 3.0 McFarland standard and undergone a microscope observation. E-test strips (bioMerieux, Marcy l’Etoile, France) were aseptically placed onto the dried surfaces of the inoculated plates for 72 h in order to determine the MIC of the antibiotics under study. Interpretation of susceptibility test results was performed in accordance with the European Committee on Antimicrobial Susceptibility Testing (EUCAST 2020) recommendations [13]. In order to define strains resistance the following MIC breakpoints were used: S ≤ 0.125 µg/mL and R > 0.125 µg/mL for AMX; S ≤ 0.25 µg/mL and R > 0.5 µg/mL, respectively, for CLA; S ≤ 1 µg/mL and R > 1 µg/mL for both TE and LEV; S ≤ 8 µg/mL and R > 8 µg/mL for MZ. Two quality control reference strains were used throughout the testing: *Hp* ATCC43504 and *Hp* RD26. Moreover, we compared four antibiotic combinations among MZ-CLA-LEV (MZ + CLA, MZ + LEV, CLA + LEV, MZ + CLA + LEV) and a single combination MZ + TE which is used in BQT recommended by Maastrich V/Florence Consensus Report (2017) [9].

### 4.3. Genotyping Susceptibility Method

Genotypic susceptibility testing was performed directly on the gastric biopsies for CLA and LEV identifying the mutations on 23S rRNA and on *gyrA* genes conferring resistance to CLA and LEV, respectively. The method used to detect the point mutations in CLA and LEV was reported elsewhere [35,42]. In brief DNA from gastric tissue samples was extracted using a DNA extraction kit (QIAamp DNA Mini Kit Qiagen Hilden, Nehren Germany) in line with manufacturer’s instructions for rapid purification of high-quality, ready-to-use DNA. Aliquots of 50 µL were used for PCR amplification. DNA regions involved in acquired clarithromycin or fluoroquinolone resistance were selectively replicated in an amplification reaction. In the following step, the amplicons were chemically denatured, since detection on the DNA strip was done using single-stranded DNA. The DNA strip is coated with highly specific probes (DNA probes) which are complementary to selectively amplified nucleic acid sequences (wild type and mutant probes). The single-stranded amplicon binds specifically to the analog probe during hybridization, while non-specifically bound amplicons are removed in subsequent washing steps. The amplified sample was hybridized, and the hybridization forms were detected by enzyme immunoassay upon addition of conjugate and substrate solution [42]. The 23S rRNA gene with its mutations A2143G, A2142C, A2142G encoding for resistance to CLA and mutations in N87K, D91N, D91G and D91Y codons in *gyrA* gene encoding for fluoroquinolone resistance were detected by a commercial reverse hybridization assay (GenoType^®^ HelicoDR test–Molecular Genetic *Hp*, Diagnostics HAIN Life Science, Nehren, Germany). This test, including different strips, is a molecular diagnostic method for easy and simultaneous detection of frequent point mutations responsible for CLA and LEV resistance. Validation control bands are designed on the strips as well. Strips are interpreted following the directions of the manufacturer. Simultaneous detection of mixed infections or heterogeneous strains was also possible with this test.

### 4.4. Therapy

Patients were treated following the guidelines with bismuth-containing quadruple therapy (Proton Pump Inhibitor, Bismuth, MZ, TE) [43]. We used TE in BQT instead of AMX in order to overcome the problem of allergic subjects. *Hp* eradication rate was identified by a validated ^13^C-urea breath test three months after therapy completion [44]. The cut-off value used was <3.5%. This cut-off has been validated in our department with sensitivity and specificity of 97% and 98%, respectively [45]. No probiotics were given to the patients together with BQT.

### 4.5. Statistical Analyses

Frequencies and percentages were used to describe the antibiotic resistance rate of *Hp* isolates. Chi-square test was utilized to compare the difference between PCR methods and E-test in CLA and LEV. A *p*-value < 0.05 was considered statistically significant.

## 5. Conclusions

All in all, we showed that in our population the resistance to MZ, CLA, and LEV, as well as multiple resistance to these antibiotics, resulted as being high. The emergence of PCR method provides a quick, convenient way to guide tailored therapy in clinical practice of *Hp* [46]. In this situation, without performing the susceptibility tests, which are time-consuming and expensive, the gastroenterologists could establish a suitable treatment for *Hp* infected patients who, in any case, underwent a gastroscopy. The quadruple therapy with proton pump inhibitor, bismuth and a combination of two antibiotics, specifically MZ and TE then unaffected by CLA and LEV resistance, turns out to be appropriate in our patients resulting in an effective *Hp* eradication rate. This treatment is also recommended by the Maastricht Consensus Group [9].

## Figures and Tables

**Table 1:** MIC_50_ and MIC_90_ values through the E-test method for the 80 *Helicobacter pylori* strains.

Antibiotics	MIC Values (µg/mL)					Resistant Strains (*n*, %)
	MIC Breakpoints		MIC_50_	MIC_90_	MIC Ranges	
	≤S	R				
Metronidazole	8	8	9–32	128	≤1–256	50 (61.6)
Clarithromycin	0.25	0.5	≤8	64	≤0.12–128	28 (35)
Levofloxacin	1	1	≤0.25	16	≤0.12–32	12 (15)
Tetracycline	1	1	≤0.25	≤1	≤0.12–≤8	2 (2.5)
Amoxicillin	0.12	0.12	≤0.12	≤0.12	≤0.12–≤1	1 (1.2)

Notes: The antibiotic breakpoints are calculated following EUCAST 2020 (European Committee on Antimicrobial Susceptibility testing). MIC, Minimum Inhibitory Concentration.

**Table 2:** *Hp* resistance to clarithromycin and levofloxacin: Comparison between real-time PCR and E-test method.

*Clarithromycin (CLA) Resistance* *(n = 80)*			*p*-Value
	PCR	E-Test	0.417
Resistance rate, *n* (%)	34 (42.5)	28 (35)	
23S rRNA genes			
−A 2143G	24	20 (MIC 4–128 µg/mL)	
−WT + A 2143G	10 *	8 (MIC >0.5–≤4 µg/mL)	
***Levofloxacin (LEV) Resistance*** ***(n = 80)***			***p*-Value**
	PCR	E-Test	0.036
Resistance rate, *n* (%)	24 (30)	12 (15)	
*gyrA* mutations			
−Codon N87 K	18	8 (MIC 4–32 µg/mL)	
−Codon D91 G	6	4 (MIC >1–≤4 µg/mL)	

The breakpoints (EUCAST 2020) were the following: S ≤ 0.25 µg/mL-R > 0.5 µg/mL for clarithromycin and S ≤ 1 µg/mL-R > 1 µg/mL for levofloxacin. * refers to mixed infections.

## Data Availability

The data supporting the results reported in the manuscript are available from the corresponding author on reasonable request.

## References

[1] Atherton J.C., Peek R.M., Tham K.T., Cover T.L., Blasér M.J. (1997). Clinical and pathological importance of heterogeneity in vacA, the vacuolating cytotoxin gene of *Helicobacter pylori*. Gastroenterology.

[2] Liu D.S., Wang Y.H., Zeng Z.R., Zhang Z.Y., Lu H., Xu J.M., Du Y.Q., Li Y., Wang J.B., Xu S.P. (2018). Primary antibiotic resistance of *Helicobacter pylori* in Chinese patients: A multiregion prospective 7-year study. Clin. Microbiol. Infect..

[3] Nahar S., Mukhopadhyay A.K., Khan R., Ahmad M.M., Datta S., Chattopadhyay S., Dhar S.C., Sarker S.A., Douglas E., Berg E. (2004). Antimicrobial Susceptibility of *Helicobacter pylori* Strains Isolated in Bangladesh. J. Clin. Microbiol..

[4] Fiorini G., Zullo A., Saracino I.M., Pavoni M., Vaira D. (2018). Antibiotic resistance pattern of *Helicobacter pylori* strains isolated in Italy during 2010–2016. Scand. J. Gastroenterol..

[5] Palmitessa V., Monno R., Panarese A., Cuppone R., Burattini O., Marangi S., Curlo M., Fumarola L., Petrosillo A., Parisi A. (2020). Evaluation of Antibiotic Resistance of *Helicobacter pylori* Strains Isolated in Bari, Southern Italy, in 2017–2018 by Phenotypic and Genotyping Methods. Microb. Drug Resist..

[6] Thung I., Aramin H., Vavinskaya V., Gupta S., Park J.Y., Crowe S.E., Valasek M.A. (2016). Review article: The global emergence of *Helicobacter pylori* antibiotic resistance. Aliment. Pharmacol. Ther..

[7] Selgrad M., Meissle J., Bornschein J., Kandulski A., Langner C., Varbanova M., Wex T., Tammer I., Schlüter D., Malfertheiner P. (2013). Antibiotic susceptibility of *Helicobacter pylori* in central Germany and its relationship with the number of eradication therapies. Eur. J. Gastroenterol. Hepatol..

[8] Bluemel H., Goelz B., Goldman J., Gruger J., Hamel H., Loley K., Ludolph T., Meyer J., Miehlke S., Mohr A. (2020). Antimicrobial resistance of *Helicobacter pylori* in Germany, 2015–2018. Clin. Microbiol. Infect..

[9] Malfertheiner P., Megraud F., O’Morain C.A., Gisbert J.P., Kuipers E.J., Axon A.T., Bazzoli F., Gasbarrini A., Atherton T., Graham D.Y. (2017). European *Helicobacter* and Microbiota Study Group and Consensus panel. Management of *Helicobacter pylori* infection-the Maastricht V/Florence Consensus Report. Gut.

[10] Wueppenhorst N., Stueger H.P., Kist M., Glocker E.O. (2013). High secondary resistance to quinolones in German *Helicobacter pylori* clinical isolates. J. Antimicrob. Chemother..

[11] Andrews J.M. (2001). Determination of minimum inhibitory concentrations. J. Antimicrob. Chemother..

[12] Versalovic J., Shortridge D., Kibler K., Griffy M.V., Beyer J., Flamm R.K., Tanaka S.K., Graham D.Y., Go M.F. (1996). Mutations in 23S rRNA are associated with clarithromycin resistance in *Helicobacter pylori*. Antimicrob. Agents Chemother..

[13] European Committee on Antimicrobial Susceptibility Testing (2020). Breakpoint Tables for Interpretation of MICs and Zone Diameters. Version 10.0. https://www.eucast.org/fileadmin/src/media/PDFs/EUCAST_files/Breakpoint_tables/v_10.0_Breakpoint_Tables.pdf.

[14] Balakrishna J.P., Filatov A. (2013). Coccoid Forms of *Helicobacter pylori* Causing Active Gastritis. Am. J. Clin. Pathol..

[15] Kanizaj T.F., Katicic M., Skurla B., Ticak M., Plecko V., Kalenic S. (2009). *Helicobacter pylori* eradication therapy success regarding different treatment period based on clarithromycin or metronidazole triple-therapy regimens. Helicobacter.

[16] Peretz A., Paritsky M., Nasser O., Brodsky D., Glyatman T., Segal S., On V. (2014). Resistance of *Helicobacter pylori* to tetracycline, amoxicillin, clarithromycin and metronidazole in Israel children and adults. J. Antibiot..

[17] Ghotaslou R., Leylabadlo H.E., Asl Y.M. (2015). Prevalence of antibiotic resistance in *Helicobacter pylori*: A recent literature review. World J. Methodol..

[18] Yi H., Yin Z., Lu N.H. (2020). Recent progress of *Helicobacter pylori* treatment. Chin. Med. J. (Engl.).

[19] Gao W., Cheng H., Hu F., Li J., Wang L., Yang G., Xu L., Zheng X. (2010). The evolution of *Helicobacter pylori* antibiotics resistance over 10 years in Beijing, China. Helicobacter.

[20] Li L., Ke Y., Yu C., Li C., Yang N., Zhang J., Li Y. (2017). Antibiotic resistance of *Helicobacter pylori* in Chinese children: A multicenter retrospective study over 7 years. Helicobacter.

[21] Horiki N., Omata F., Uemura M., Suzuki S., Ishii N., Iizuka Y., Fukuda K., Fujita Y., Katsurahara M., Imoto I. (2009). Annual change of primary resistance to clarithromycin among *Helicobacter pylori* isolates from 1996 through 2008 in Japan. Helicobacter.

[22] Choi Y.I., Jeong S.H., Chung J.W., Park D.K., Kim K.O., Kwon K.A., Kim Y.J., So S., Lee J.H., Jeong J.Y. (2019). Rifabutin and Furazolidone Could Be the Candidates of the Rescue Regimen for Antibiotic-Resistant, *H. pylori* in Korea. Can. J. Infect. Dis. Med. Microbiol..

[23] Bouihat N., Burucoa C., Benkirane A., Seddik H., Sentissi S., Al Bouzidi A., Elouennas M., Benouda A. (2017). *Helicobacter pylori* primary antibiotic resistance in 2015 in Morocco: A phenotypic and genotypic prospective and multicenter study. Microb. Drug Resist..

[24] Shiota S., Reddy R., Alsarraj A., El-Serag H.B., Graham D.Y. (2015). Antibiotic resistance of *Helicobacter pylori* among male United States veterans. Clin. Gastroenterol. Hepatol..

[25] Mitui M., Patel A., Leos N.K., Doern C.D., Park J.Y. (2014). Novel *Helicobacter pylori* sequencing test identifies high rate of clarithromycin resistance. J. Pediatr. Gastroenterol. Nutr..

[26] Saracino I.M., Zullo A., Holton J., Castelli V., Fiorini G., Zaccaro C., Ridola L., Ricci C., Gatta L., Vaira D. (2012). High prevalence of primary antibiotic resistance in *Helicobacter pylori* isolates in Italy. J. Gastrointest. Liver Dis..

[27] Rhie S.Y., Park J.Y., Shin T.S., Kim J.W., Kim B.J., Kim J.G. (2020). Discovery of a novel mutation in DNA gyrase and changes in the fluoroquinolone resistace of *Helicobacter pylori* over a 14-year period: Single centre study in Korea. Antibiotics.

[28] Gerrits M.M., Van der Wouden E.J., Bax D.A., Van Zwet A.A., Van Vliet A.H.M., De Jong A., Kusters J.G., Thijs J.C., Kuipers E.J. (2004). Role of the rdxA and frxA genes in oxygen dependent metronidazole resistance of *Helicobacter pylori*. J. Med. Microbiol..

[29] Torres J., Camorlinga-Ponce M., Perez-Perez G., Madrazo-De la Garza A., Dehesa M., González-Valencia G., Muñoz O. (2001). Increasing multidrug resistance in *Helicobacter pylori* strains isolated from children and adults in Mexico. J. Clin. Microbiol..

[30] Khademi F., Poursina F., Hosseini E., Akbari M., Safaei H.G. (2015). *Helicobacter pylori* in Iran: A systematic review on the antibiotic resistance. Iran. J. Basic Med. Sci..

[31] Saeed A., Bosch A., Bettiol M., Nossa González D.L., Erben M.F., Lamberti Y. (2018). Novel Guanidine Compound against Multidrug-Resistant Cystic Fibrosis-Associated Bacterial Species. Molecules.

[32] Chimenti F., Bizzarri B., Bolasco A., Secci D., Chimenti P., Carradori S., Granese A., Rivanera D., Lilli D., Scaltrito M. (2006). Synthesis and in vitro selective anti-*Helicobacter pylori* activityofN-substituted-2-oxo-2H-1-benzopyran-3-carboxamides. Eur. J. Med. Chem..

[33] Makipour K., Friedenberg F.K. (2011). The potential role of N-acetylcysteine for the treatment of *Helicobacter pylori*. J. Clin. Gastroenterol..

[34] Matsumoto H., Shiotani A., Katsumata R., Fujita M., Nakato R., Murao T., Ishii M., Kamada T., Haruma K., Graham D.Y. (2016). *Helicobacter pylori* eradication with proton pump inhibitors or potassium-competitive acid blockers: The effect of clarithromycin resistance. Dig. Dis. Sci..

[35] Binyamin D., Pastukh N., On A., Paritsky M., Peretz A. (2017). Phenotypic and genotypic correlationas expressed in *Helicobacter pylori* resistance to clarithromycin and fuoroquinolones. Gut Pathog..

[36] Jyh M.L., Chang C.Y., Sheng W.H., Wang Y.C., Chen M.J., Lee Y.C., Hung H.W., Chian H., Chang S.C., Wu M.S. (2011). Genotypic Resistance in *Helicobacter pylori* Strains Correlates with Susceptibility Test and Treatment Outcomes after Levofloxacin- and Clarithromycin-Based Therapies. Antimicrob. Agents Chemother..

[37] Lee J.W., Kim N., Nam R.H., Park J.H., Choi Y.J., Kim J.M., Kim J.S., Jung H.C. (2014). GenoType HelicoDR test in the determination of antimicrobial resistance of *Helicobacter pylori* in Korea. Scand. J. Gastroenterol..

[38] Graham D.Y., Lee Y.C., Wu M.S. (2014). Rational *Helicobacter pylori* therapy: Evidence-based medicine rather than medicine-based evidence. Clin. Gastroenterol. Hepatol..

[39] Uotani T., Graham D.Y. (2015). Diagnosis of *Helicobacter pylori* using the rapid urease test. Ann. Transl. Med..

[40] Tonkic A., Vucovic J., Cindro P.V., Pisac V.P., Tonkic M. (2017). Diagnosis of *Helicobacter pylori* infection. A short review. Wien. Klin. Wochenschr..

[41] Mascellino M.T., Oliva A., De Angelis M., Pontone S., Porowska B. (2018). *Helicobacter pylori* infection: Antibiotic resistance and eradication rate in patients with gastritis showing previous treatment failures. New Microbiol..

[42] Miendje Deyi V.Y., Burette A., Bentatou Z., Maaroufi Y., Bontems P., Lepage P., Reynders M. (2011). Practical use of GenoType^®^ HelicoDR, a molecular test for *Helicobacter pylori* detection and susceptibility testing. Diagn. Microbiol. Infect. Dis..

[43] Tursi A., Di Mario F., Franceschi M., De Bastiani R., Elisei W., Baldassarre G., Ferronato A., Grillo S., Landi S., Zamparella M. (2017). New bismuth-containing quadruple therapy in patients infected with *Helicobacter pylori*: A first Italian experience in clinical practice. Helicobacter.

[44] Gisber J.P., Pajares J.M. (2004). Review article: ^13^ C-urea breath test in the diagnosis of *Helicobacter pylori* infection—A critical review. Aliment. Pharmacol. Ther..

[45] Capurso G., Martino G., Grossi C., Delle Fave D. (2000). Hypersecretory duodenal ulcer and *Helicobacter pylori* infection: A four-year follow-up study. Dig. Liver Dis..

[46] Chen Q., Long X., Ji Y., Liang X., Li D., Gao H., Xu B., Liu M., Chen Y., Sun Y. (2019). Randomized controlled trial: Susceptibility-guided therapy versus empiric bismuth quadruple therapy for first-line *Helicobacter pylori* treatment. Aliment. Pharmacol. Ther..

